# Tyrosine Kinase Inhibitors Regulate OPG through Inhibition of PDGFRβ

**DOI:** 10.1371/journal.pone.0164727

**Published:** 2016-10-13

**Authors:** Susannah O’Sullivan, Mei Lin Tay, Jian-Ming Lin, Usha Bava, Karen Callon, Jillian Cornish, Dorit Naot, Andrew Grey

**Affiliations:** 1 Department of Pharmacology, University of Auckland, Auckland, New Zealand; 2 Department of Medicine, University of Auckland, Auckland, New Zealand; Universite de Nantes, FRANCE

## Abstract

Nilotinib and imatinib are tyrosine kinase inhibitors (TKIs) used in the treatment of chronic myeloid leukemia (CML) and gastrointestinal stromal tumors (GIST). *In vitro*, imatinib and nilotinib inhibit osteoclastogenesis, and in patients they reduce levels of bone resorption. One of the mechanisms that might underlie these effects is an increase in the production of osteoprotegerin (OPG). In the current work we report that platelet-derived growth factor receptor beta (PDGFRβ) signaling regulates OPG production in vitro. In addition, we have shown that TKIs have effects on RANKL signaling through inhibition of the PDGFRβ and other target receptors. These findings have implications for our understanding of the mechanisms by which TKIs affect osteoclastogenesis, and the role of PDGFRβ signaling in regulating osteoclastogenesis. Further studies are indicated to confirm the clinical effects of PDGFRβ-inhibitors and to elaborate the intracellular pathways that underpin these effects.

## Introduction

Imatinib is an orally active tyrosine kinase inhibitor (TKI) which is established as a first-line therapy for patients with bcr-abl positive chronic myeloid leukemia (CML) [1, 2]. As well as inhibiting bcr-abl, imatinib inhibits all abl tyrosine kinases (TKs) [3], the platelet-derived growth factor (PDGF) receptors α and β [4], c-kit [5] and c-fms at therapeutic concentrations [6]. It is commonly used in the treatment of gastrointestinal stromal tumors (GIST) in which there are mutations of the *KIT* gene [7, 8]. Nilotinib is a TKI developed to manage imatinib-resistance in patients with CML, and inhibits similar molecular targets to imatinib, although is a more potent inhibitor of bcr-abl [9–11]. Both TKIs exhibit off-target effects due to inhibition of their molecular targets in healthy tissues [12, 13]. Studies published by our group and others suggest that imatinib and nilotinib affect bone and calcium metabolism [10, 14–30]. With regards to effects on osteoclasts, *in vitro* they decrease osteoclast formation and function by both direct and indirect, stromal-cell dependent mechanisms [10, 14, 16, 27, 29, 31]. In patients with CML they reduce levels of the bone resorption marker β-C-terminal telopeptide of type I collagen (βCTX) [22, 24, 25, 29, 32, 33], with a neutral or possibly beneficial effect on the skeleton [20, 22, 23, 26, 29, 33].

More recently, interest has developed in the potential role of these drugs in the management of malignant and non-malignant bone diseases, as a result of anti-resorptive activity [14, 27, 34, 35]. The majority of attention has focused on the direct inhibition of osteoclastogenesis by TKIs. This effect has been attributed to inhibition of the c-Fms receptor [10, 14, 27, 35, 36], although PDGFRβ inhibition by trapidil inhibits osteoclastogenesis by suppressing receptor activator of nuclear factor κB (RANK) ligand-induced nuclear factor of activated T-cells (NFAT)1c expression in osteoclast precursors [37]. We have shown that an important mechanism by which imatinib and nilotinib have an inhibitory effect on osteoclastogenesis is indirectly through an increase in the expression and secretion of osteoprotegerin (OPG) [16, 29]. OPG acts as a decoy receptor that binds to RANKL and blocks its interaction with RANK thus inhibiting osteoclast development [38]. Both imatinib and nilotinib increase gene expression and protein secretion of OPG in stromal and osteoblastic cells [16, 29]. Patients treated with imatinib have been found to have an increased OPG/RANKL ratio [17]. The mechanism by which TKIs stimulate production of OPG is not known, however a potential candidate for mediating these effects is the PDGFRβ, as we have previously shown that inhibition of the PDGFRβ is the main mechanism by which TKIs affect growth and maturation of osteoblastic cells *in vitro* [16, 29]. In the current work, we have investigated the role that inhibition of PDGFRβ plays in the effects of imatinib and nilotinib to increase OPG production and indirectly inhibit osteoclastogenesis.

## Materials and Methods

### Media and Reagents

Minimum essential media (MEM), minimum essential media α modification (αMEM), and Dulbecco’s minimum essential media (DMEM) powder, Opti-MEM^®^, sodium pyruvate (NaP), fetal bovine serum (FBS) and Penicillin/Streptomycin mixture (10,000U/mL) were purchased from Gibco BRL (ThermoFisher Scientific, Waltham, MA). L-ascorbic acid-2-phosphate (AA2P), bosutinib and puromycin dihydrochloride were purchased from Sigma-Aldrich Co. (St. Louis, MO). Imatinib mesylate and nilotinib were supplied by Novartis Pharma AG (Basel, Switzerland). Rat PDGF-BB was purchased from R&D Systems (Minneapolis, MN). Polybrene was purchased from Santa Cruz Biotechnology (Dallas, TX). Lipofectamine^®^ 2000 Transfection Reagent was purchased from Life Technologies (ThermoFisher Scientific).

### Primary Cell Culture

E20 Wistar fetal rats (sourced from the VJU research unit and approved by the University of Auckland Animal Ethics Committee) were euthanised by rapid decapitation and the calvariae excised and the frontal and parietal bones, free of suture and periosteal tissue, were collected. The calvariae bones were sequentially digested using collagenase and the osteoblast-like cells from digests 3 and 4 were collected, pooled, and washed. Cells were grown in T75 flasks in DMEM supplemented with 10% FBS and 5ug/ml AA2P for 2 days and then changed to MEM supplemented with 10% FBS and 5ug/ml AA2P and the cells grown to 90% confluence. The osteoblast-like character of these cells has been established by demonstration of high levels of alkaline phosphatase activity and osteocalcin production [39] and a sensitive adenylyl cyclase response to parathyroid hormone and prostaglandin E_2_ [40].

Four to 6-week-old Swiss male mice (sourced from the VJU research unit and approved by the University of Auckland Animal Ethics Committee) were sacrificed by cervical dislocation while under halothane or CO_2_ anaesthesia. Femora and tibiae were aseptically removed and dissected free of adhering tissues. The epiphyses were cut off with a scalpel blade and the marrow cavity was flushed with α-minimum essential medium (αMEM) using a syringe with a 23G needle. The marrow cells were collected in a 50mL centrifuge tube, spun at 1200 rpm for 2 min, and washed with αMEM /10% fetal bovine serum. Marrow cells were then cultured for 2 h in 90 mm Petri dishes. After 2 h, non-adherent cells were collected, spun at 1200 rpm for 2 min, washed with αMEM/15% FBS, and seeded at 1.0 x 10^6^cells/ml in 6 well plates (2.5ml/well).

Murine stromal ST2 cells (St Vincent’s Institute, Melbourne, Australia), and murine pre-osteoblastic MC3T3-E1 cells (ATCC, Cryosite Distribution, Lane Cove, NSW, Australia) were maintained in standard cell culture conditions.

All protocols involving use of animals have been approved by the University of Auckland Animal Ethics Committee.

### Osteoblast Production of OPG

In experiments designed to test the effects of imatinib, nilotinib, bosutinib and PDGF-BB on OPG expression and secretion, ST2 and primary rat osteoblastic cells were cultured overnight in 5% FBS, then the media changed to 1% FBS at the time of addition of the drug as previously described [16]. RNA and conditioned media were collected at baseline and after 8, 24, 48 and 72 hours for analysis of expression of the target genes of interest and protein secretion. Assays in murine bone marrow cells were performed as previously described [29] and cell pellets were collected at baseline, 24, 72 and 120 hours after addition of drugs for analysis of expression of the target genes of interest. OPG was measured in conditioned media using the murine osteoprotegerin/TNFRSF11B DuoSet (R&D Systems), according to the manufacturer’s instructions. Gene expression was analyzed as detailed below.

### Analysis of Gene Expression

Total cellular RNA was extracted from cultured cells and purified using RNeasy mini kit (Qiagen, Venlo, Netherlands). Genomic DNA was removed using RNase-free DNase set (Qiagen). Reverse transcription was carried out using SuperScript III (Life Technologies, ThermoFisher Scientific) as previously described [41], and cDNA was used for real-time PCR. Multiplex PCR was performed with FAM^™^-labeled TaqMan assays specific for the genes of interest, and VIC^®^-labeled 18S rRNA endogenous control TaqMan assays according to the company’s instructions, using ABI PRISM 7900HT Sequence Detection System (Applied Biosystems, ThermoFisher Scientific). The primer-probe sets were purchased from Applied Biosystems (ThermoFisher Scientific). Samples were assayed in duplicate or triplicate. The relative level of mRNA expression was determined using the ΔΔCt calculation method as previously described [41]. Expression data were normalized to the control value at the earliest time point assayed.

### RNA Interference

Short-term RNA interference (“gene-silencing”) was performed as previously described [29] with Stealth RNAi^™^ probes specific to murine *PDGFRB*, *PDGFRA*, *ABL-1* or a GC control sequence (ThermoFisher Scientific). Cell pellets and conditioned media were collected at 6, 24, 48, 72 (and in some cases 120 and 168) hours after transfection for analysis of gene and protein expression. Longer-term RNA interference was achieved using lentiviral short hairpin RNA (shRNA) delivery. In order to assess the effects of silencing *PDGFRB* on osteoblast differentiation, in addition to OPG production, pre-osteoblastic cell MC3T3 E1 cells were used. Briefly, MC3T3-E1 cells were seeded in a 48-well plate at a density of5x10^3^ cells/well in 10% FBS/MEM/sodium pyruvate. After 24h, when cells were 50% confluent, *PDGFRB* shRNA, control shRNA-A and copGFP control lentiviral particles (Santa Cruz Biotechnology) were transduced into the cells with addition of 10μg/mL polybrene at a multiplicity of infection (MOI) of 5. Culture medium was replaced 24h after transduction. The effectiveness of the transduction was verified using fluorescence from the copGFP control transduced cells. After a further 5 days, 2.5ug/mL puromycin dihydrochloride was added, and after further incubation of 4 days colonies with puromycin resistance were identified and isolated. *PDGFRB* and PDGFRβ expression were assessed using real-time PCR and immunoblotting respectively. RNA was collected for 4 subcultures for analysis of expression of the target genes of interest.

### Immunoblotting

ST2 cells were seeded and treated as per RNA interference. After 30 hours, the treatment medium was aspirated and the cells were washed in ice-cold PBS and directly lysed in 2 x SDS-PAGE loading buffer. Lysates were homogenised, boiled, centrifuged at 1200rpm for 1 minute at room temperature, then stored at -20°C until analysed. Protein samples were resolved by 4–20% Mini-Protean TGX Gel (Bio-Rad, Hercules, CA) and transferred to polyvinylidene fluoride (PVDF) membranes (Bio-Rad) and blocked with 5% (w/v) non-fat milk powder in T-TBS buffer (1% Tween in 20mM TRIS-HCl/1237mM NaCl; pH 7.6). The membranes were incubated overnight at 4°C with either rabbit monoclonal anti-PDGFRβ antibody (C82A3, Cell Signalling Technology, Danvers, MA; 1:1000) or mouse monoclonal antibody against alpha-tubulin (T5168, Sigma-Aldrich; 1:500) for internal loading control. Immunoreactivities were visualised by incubation for 1 hour at room temperature with peroxidase-conjugated goat anti-rabbit IgG (A0545, Sigma-Aldrich, 1:10,000) or anti-sheep secondary antibody and development by chemiluminescence using Amersham ECL Plus Western Blotting Detection Reagents (GE Healthcare, Buckinghamshire, UK).

In separate experiments, control shRNA-A transduced and *PDGFRB* shRNA transduced MC3T3-E1 cells were cultured in 6-well tissue culture plates and cell lysates were collected for 4 subcultures. When subcultures reached confluence, the culture medium was aspirated, the cells were washed in ice-cold PBS and then scraped in ice-cold RIPA lysis buffer [25mM Tris-HCl pH 7.6, 150mM NaCl, 1% NP-40, 1% sodium deoxycholate, 0.1% SDS] (Pierce Biotechnology, ThermoFisher Scientific Inc.) containing a cocktail of protease inhibitors (cOmplete, Mini Protease Inhibitor Cocktail Tablets; Roche Diagnostics, Mannheim, Germany). The lysates were briefly vortexed, clarified by centrifugation at 12,000 rpm for 20 minutes at 4°C, then stored at −80°C until analyzed. The protein content of the cell lysates was measured using a Pierce BCA Protein kit (ThermoFisher Scientific Inc.) Lysates (2μG/well) were then subjected to 4–15% precast polyacrylamide gels (Bio-Rad, Hercules, CA), transferred to PVDF membranes and blocked with 5% (w/v) non-fat milk powder in TBS-T buffer (1% Tween-20 in 50mM TRIS–HCl/150 mM NaCl; pH 7.4) before immunoblotting overnight at 4°C with an antibody to PDGFRβ (C82A3, Cell Signalling Technology, Danvers, MA, 1:200). Incubation with the horseradish peroxidase-conjugated anti-rabbit secondary antibody (A0545, Sigma-Aldrich, 1:2000) was for 1 hour at room temperature, and bands were visualized with ECL. As a control for protein loading, the same filters were stripped and re-probed with an antibody to beta-actin (A5441, Sigma-Aldrich, 1:10,000) followed by incubation with rabbit anti-mouse IgG (A9044, Sigma-Aldrich, 1:20,000)

### Statistical Analyses

Data were analyzed using GraphPad Prism (v5.04) (GraphPad Software, San Diego, CA). Data from experiments evaluating multiple time points or drug/peptide concentrations were analyzed by two-way ANOVA with Bonferroni’s post-hoc test.

## Results

### Tyrosine Kinase Inhibitors that inhibit the PDGFRβ increase OPG levels

**Fig 1:**
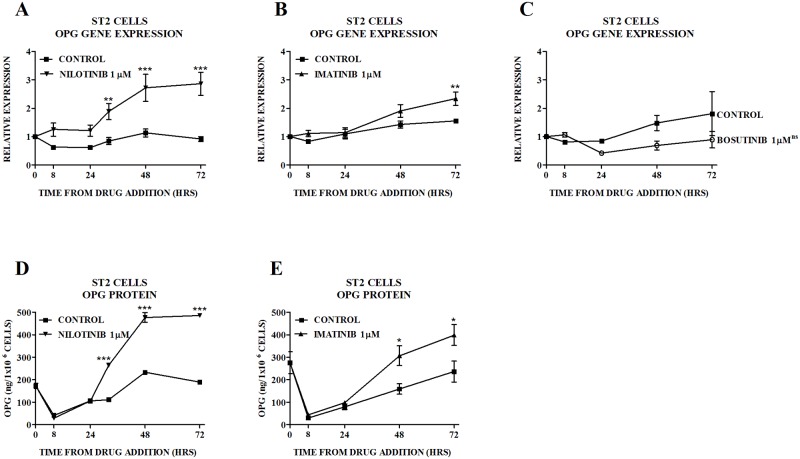
Effect of TKIs on OPG in ST2 Cells. Effect of nilotinib on OPG (A) gene expression and (D) protein production. Figs 1A and D have previously been published [29] (S2 File) and are reproduced for illustrative purposes with permission (S1 File). Effect of imatinib on OPG (B) gene expression and (E) protein production. (C) Effect of bosutinib on OPG gene expression. Gene expression is quantitated relative to the baseline control value. In the case of ST2 cells treated with bosutinib, individual time-points were not significantly different from control, thus the p-value for the overall difference between the treatment group and the control group is shown on Fig 1E. Data are mean ± SEM. ^ns^not significant, *p<0.05, **p<0.01, ***p<0.001 vs untreated control.

We have previously shown that imatinib increases OPG gene expression (primary rat osteoblasts) and protein production (ST2 cells) [16], and that nilotinib increases OPG gene expression (ST2 cells, murine bone marrow) and protein production (ST2 cells) (Fig 1A and 1D, reproduced for illustrative purposes with permission (S1 File) [29] (S2 File). In the current study in order to investigate the effects of imatinib on OPG production, we performed biological repeats that assessed changes in OPG gene expression (Fig 1C) and protein production (Fig 1D) in ST2 cells treated with imatinib, and showed that gene expression leads to a corresponding increase in protein secretion. In contrast, bosutinib which does not inhibit the PDGFR (IC_50_ > 1000μM) or c-kit, did not have an effect on OPG gene expression (Fig 1C) providing evidence that PDGFRβ inhibition may mediate the effects of nilotinib (IC_50_ for PDGFR phosphorylation 72) and imatinib (IC_50_ for PDGFR phosphorylation 74) on.

**Fig 2:**
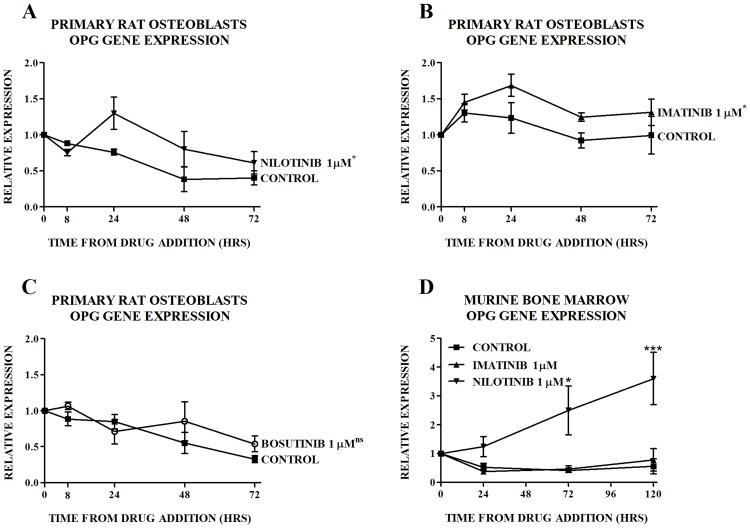
Effect of TKIs on Expression of OPG in Primary Cells. Effect of (A) nilotinib, (B) imatinib, and (C) bosutinib on expression of OPG mRNA in primary rat osteoblasts. (D) Effect of nilotinib and imatinib on expression of OPG mRNA in murine bone marrow. Gene expression is quantitated relative to the baseline control value. Data are mean ± SEM. In the case of primary rat osteoblasts treated with nilotinib, imatinib and bosutinib, individual time-points were not significantly different from control, thus the p-value for the overall difference between the treatment group and the control group is shown on Figs 2A-C. ^ns^not significant, *p<0.05, ***p<0.001 vs untreated control at each time point.

OPG gene production. We then investigated and compared the effects of imatinib, nilotinib and bosutinib on OPG gene expression in primary osteoblastic cells. We performed novel experiments to determine the effects of nilotinib and bosutinib on OPG gene expression. For purposes of comparison, we performed contemporaneous experiments with imatinib and found similar effects to those seen in ST2 cells (Fig 2A and 2B). Bosutinib did not have an effect on OPG gene expression (Fig 2C). The murine osteoprotegerin/TNFRSF11B DuoSet (R&D Systems) was not able to detect rat OPG, thus protein production was not measured. In murine bone marrow we performed biological repeats to assess the effects of imatinib and nilotinib in the same culture. In this mixed population of primary cells that includes osteoblastic precursors, in keeping with previous findings, nilotinib but not imatinib increased OPG gene expression (Fig 2D).

### Imatinib and nilotinib inhibit the effects of PDGF-BB on OPG levels

**Fig 3:**
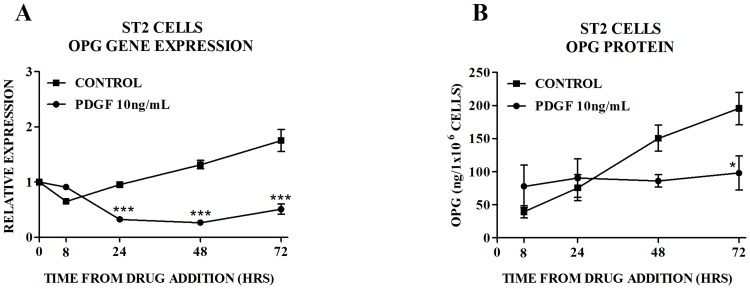
Effect of PDGF-BB (PDGF) on OPG in ST2 Cells. Effect of PDGF-BB (PDGF) on (A) expression of OPG mRNA and (B) production of OPG protein by ST2 cells. Gene expression is quantitated relative to the baseline control value. Data are mean ± SEM. *p<0.05, ***p<0.001 vs untreated control at each time point.

**Fig 4:**
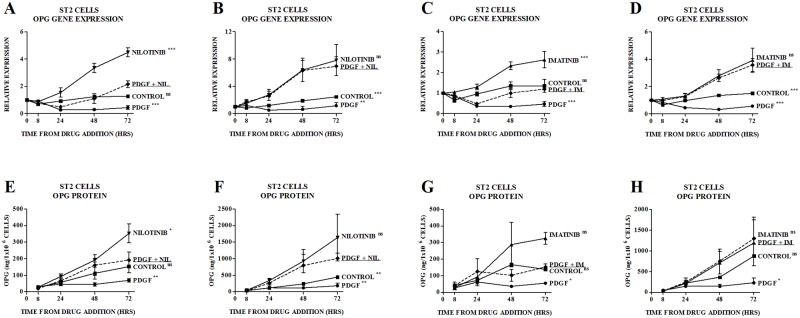
Effect of PDGF-BB (PDGF), Nilotinib and Imatinib on OPG in ST2 Cells. Partial reversal of the effect of PDGF 10ng/ml by nilotinib 0.1μM on (A) expression of OPG mRNA in ST2 cells and (E) production of OPG protein by ST2 cells. Reversal of the effect of PDGF 10ng/ml by nilotinib 1.0μM on (B) expression of OPG mRNA in ST2 cells and (F) production of OPG protein by ST2 cells. Partial reversal of the effect of PDGF 10ng/ml by imatinib 0.1μM on (C) expression of OPG mRNA in ST2 cells and (G) production of OPG protein by ST2 cells. Reversal of the effect of PDGF 10ng/ml by imatinib 1.0μM on (D) expression of OPG mRNA in ST2 cells and (H) production of OPG protein by ST2 cells. Gene expression is quantitated relative to the appropriate baseline value. Data are mean ± SEM. ^ns^not significant, *p<0.05, **p<0.01, ***p<0.001 vs PDGF/nilotinib-treated or PDGF/imatinib-treated group. NIL, nilotinib. IM, imatinib.

Given the above findings and the critical role that PDGFRβ signaling plays in mediating the osteoblastic effects of TKIs [16, 29] we investigated the effect of activation of PDGFRβ signaling on OPG production. Treatment of ST2 cells with PDGF-BB (the exclusive ligand for PDGFRβ) inhibited OPG gene expression and protein secretion (Fig 3A and 3B). Pretreatment with nilotinib 0.1μM (Fig 4A and 4E) or imatinib 0.1μM (Fig 4C and 4G) partially reversed this inhibitory effect, such that levels of OPG were similar to those seen in the control group. At a higher concentration of nilotinib (1.0 μM) (Fig 4B and 4F) and imatinib (1.0 μM) (Fig 4D and 4H), levels of OPG gene expression and protein secretion were similar to those seen with nilotinib or imatinib alone.

### Silencing of the *PDGFRB* gene increases OPG levels

**Fig 5:**
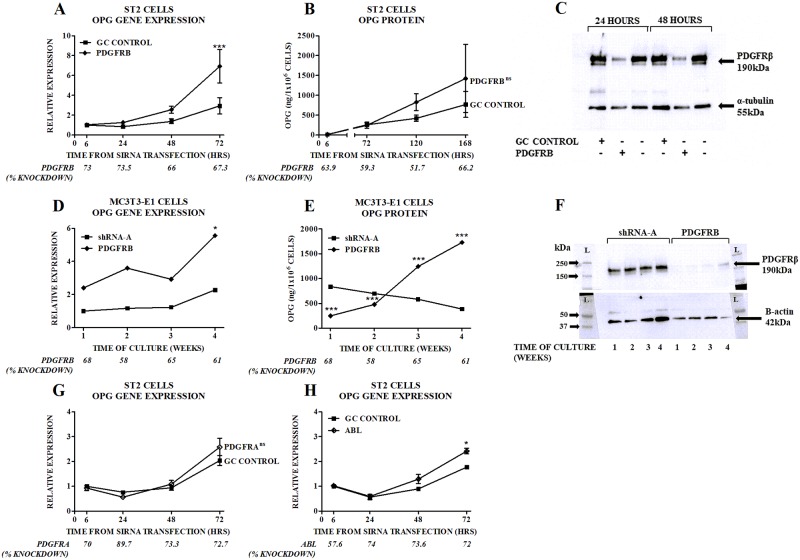
Effects of *PDGFRB* Gene Silencing on OPG in ST2 and MC3T3-E1 Cells. Effects of siRNA targeting *PDGFRB* on (A) expression of OPG mRNA in ST2 cells and (B) production of OPG protein by ST2 cells. The level of gene silencing achieved is indicated in the second row of the x-axis of each graph. Gene expression is quantitated relative to the baseline control oligo value. Data are mean ± SEM. ***p<0.001 vs control oligo. OPG protein levels were not significantly different at individual time-points between ST2 cells with *PDGFRB* gene silencing and those with control oligo, thus the p-value for the overall difference between the two groups is shown on Fig 5B. (C) Effects of siRNA targeting *PDGFRB* on PDGFRβ protein levels. The immunoblot presented is representative of at least three separate experiments. OPG gene expression (D) or protein secretion (E) in *PDGFRB* shRNA transduced MC3T3-E1 cells. The level of *PDGFRB* gene expression or protein is shown on the x-axis. Gene expression is quantitated relative to the levels in SHRNA-A control cells at baseline. Data are mean. (F) PDGFRβ protein levels in *PDGFRB* shRNA transduced MC3T3-E1 cells. Effects of siRNA targeting (G) *PDGFRA* or (H) *ABL* on expression of OPG mRNA in ST2 cells. Gene expression is quantitated relative to the baseline control oligo value. Data are mean ± SEM. * p<0.05 vs control oligo. OPG gene expression was not significantly different at individual time-points between ST2 cells with *PDGFRA* gene silencing and those with control oligo, thus the p-value for the overall difference between the two groups is shown on Fig 5G.

To directly confirm the role of the PDGFRβ in regulating OPG production we used gene silencing techniques to inhibit expression of the *PDGFRB* gene. Using short-term RNA interference, *PDGFRB* gene expression (Fig 5A and 5B, x axes labels) and PDGFRβ protein production (Fig 5C) were inhibited, resulting in a 2.5 fold increase in OPG gene expression and a lesser (2 fold) non-significant increase in protein production (Fig 5A and 5B). In MC3T3-E1 cells stably transduced with PDGFRB shRNA, longer term inhibition of *PDGFRB* gene expression (Fig 5D) and PDGFRβ protein production (Fig 5F), led to a 2.4–3 fold increase in OPG gene expression (Fig 5D) and a 2–4.5 fold increase in protein secretion (Fig 5E). We considered the possibility that inhibition by imatinib and nilotinib of one of their other target receptors may be contributing to the effects we observed. Inhibition of *PDGFRA* gene expression by 70–90% did not increase OPG gene expression (Fig 5G), and inhibition of *ABL* gene expression by more than 70% led to a 1.4 fold increase in OPG production (Fig 5H); ST2 cells do not express the KIT gene [16].

### TKIs reduce RANKL levels potentially through inhibition of PDGRβ signaling

**Fig 6:**
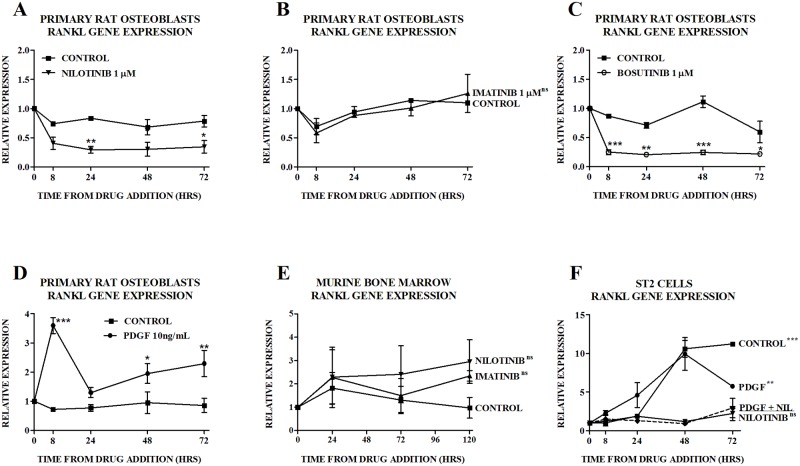
Effect of TKIs and PDGF-BB (PDGF) on RANKL. Effect of (A) nilotinib, (B) imatinib, (C) bosutinib and (D) PDGF-BB (PDGF) on expression of RANKL mRNA in primary rat osteoblasts. Gene expression is quantitated relative to the baseline control value. Data are mean ± SEM. In the case of primary rat osteoblasts treated with imatinib, individual time-points were not significantly different from control, thus the p-value for the overall difference between the treatment group and the control group is shown on Fig 6B. ^ns^not significant, *p<0.05, **p<0.01, ***p<0.001 vs vs untreated control. Effect of nilotinib and imatinib 1 μM on expression of RANKL mRNA in murine bone marrow (Fig 6E). Data are mean ± SEM. Individual time-points were not significantly different from control, thus the p-value for the overall difference between the treatment group and the control group is shown on 6E. ^ns^not significant vs untreated control. Effect of PDGF 10ng/ml and nilotinib 1.0μM on expression of RANKL mRNA in ST2 cells (Fig 6F). Gene expression is quantitated relative to the appropriate baseline value. Data are mean ± SEM. ^ns^not significant, **p<0.01, ***p<0.001 vs PDGF/nilotinib-treated group. NIL, nilotinib.

We have previously reported that imatinib has no effect on RANKL levels in bone marrow from patients treated with imatinib for 6 months [16] but RANKL mRNA expression was reduced in cultures of ST2 cells treated with nilotinib [29]. In primary rat osteoblasts, consistent with our previous findings, nilotinib reduced and imatinib had no effect on RANKL gene expression (Fig 6A and 6B respectively). The c-Src/c-Abl inhibitor, bosutinib, reduced RANKL gene expression (Fig 6C), while PDGF-BB increased RANKL gene expression (Fig 6D). In murine bone marrow, none of nilotinib, imatinib (Fig 6E) or PDGF-BB (data not shown) had an effect on levels of RANKL mRNA. In ST2 cells that underwent pretreatment with nilotinib 1.0μM, RANKL gene expression induced by PDGF-BB was similar to that seen with nilotinib alone, implying no effect of PDGF-BB (Fig 6E). Short-term RNA interference of the *PDGFRB*, *PDGFRA* or *ABL* gene in ST2 cells did not affect RANKL gene expression (data not shown).

## Discussion

Nilotinib and imatinib decrease osteoclast development and function *in vitro* [10, 14, 16, 27, 29, 31] and reduce markers of bone resorption in humans [22, 24, 25, 29, 32, 33]. These finding have created interest in a potential role for TKIs in the management of malignant and non-malignant bone lesions that result from increased osteoclast activity and excessive bone resorption [14, 27, 34, 35]. Although attention has focused on the direct inhibition of osteoclastogenesis by TKIs [10, 14, 27, 35, 36], our previous work suggested that a mechanism by which imatinib and nilotinib might inhibit osteoclastogenesis is indirect, through an increase in the expression and secretion of OPG [16, 29].

**Fig 7:**
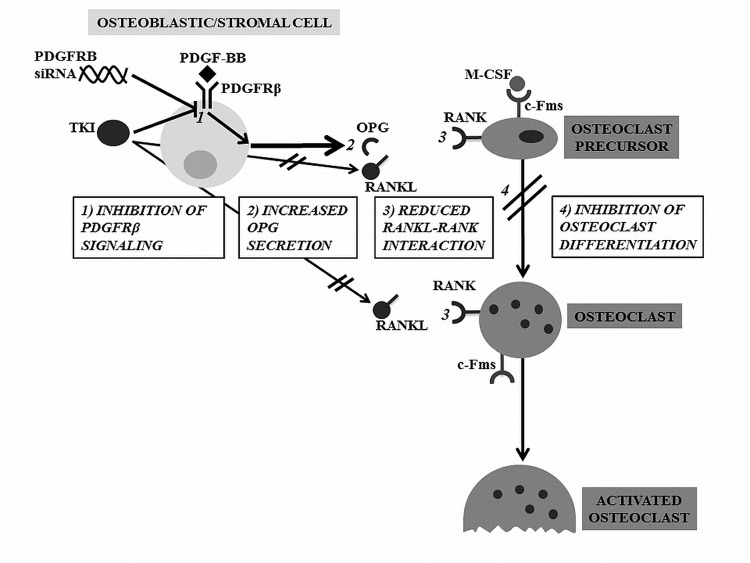
Mechanisms by Which Inhibition of PDGFRβ by TKIs or Gene Silencing Inhibits Osteoclastogenesis. TKIs or *PDGFRB* gene silencing (SiRNA) inhibit PDGFRβ signaling (1) with a resultant increase in OPG production (2). This reduces RANK-RANKL interaction (3) leading to inhibition of osteoclast differentiation (4). Additionally, TKIs reduce RANKL secretion but not through PDGFRβ-mediated mechanisms.

In the current work, we report indirect and direct evidence for a role of PDGFRβ in the effects of imatinib and nilotinib on OPG production, which may be a mechanism by which these agents inhibit osteoclastogenesis (Fig 7). Firstly, imatinib and nilotinib, which both inhibit PDGFRβ signaling increase OPG levels while bosutinib, which is a potent TKI but does not inhibit the PDGFR, did not increase OPG expression. Secondly, PDGF-BB inhibits OPG expression, and pre-treatment with either nilotinib or imatinib reverses that effect. Third, gene silencing of *PDGFRB* replicated the effects of nilotinib and imatinib to increase gene expression and protein secretion of OPG. Marginal or no effects on OPG expression were observed in response to gene silencing of *ABL* and *PDGFRA*.

Our previous work has shown that the effects of TKIs on the OPG/RANKL system are predominantly due to stimulation of OPG production, with no change in RANKL gene expression in bone marrow samples from patients treated with imatinib and a variable effect on RANKL gene expression in stromal cells treated with imatinib or nilotinib *in vitro* [16, 29]. Here we find that in primary rat osteoblasts, nilotinib and bosutinib but not imatinib inhibited RANKL, while PDGF-BB had the opposite effect. However, direct inhibition of *PDGFRB* using gene silencing did not affect RANKL gene expression. In light of these results, the contribution that modulation of RANKL expression plays in the effects of TKIs on osteoclastogenesis remains uncertain.

Overall these findings suggest that OPG expression and production by osteoblasts is regulated by PDGFRβ signaling. Further studies are indicated to confirm the clinical effects of PDGFRβ-inhibitors in the setting of activated bone resorption, and to elaborate the intracellular pathways that underpin the effects of TKIs and the PDGFRβ on OPG and RANKL.

## Supporting Information

S1 File Written Permission for Fig 1A and 1D. Written permission from the original copyright holder to publish Fig 1A and 1D in the current manuscript.
(PDF)

S2 File The skeletal effects of the tyrosine kinase inhibitor nilotinib. Source publication for Fig 1A and 1D.
(PDF)
