# Supplementary material for: Tyrosine Kinase Inhibitors Regulate OPG through Inhibition of PDGFRβ
Source: PLoS One. 2016 Oct 13;11(10):e0164727. doi: 10.1371/journal.pone.0164727 (PMC5063333; doi:10.1371/journal.pone.0164727)
Supplement: S2 File — Source publication for Fig 1A and 1D. (PDF) [file pone.0164727.s002.pdf]

Bone 49 (2011) 281–289

Contents lists available at ScienceDirect

Bone

journal homepage: www.elsevier.com/locate/bone

# The skeletal effects of the tyrosine kinase inhibitor nilotinib

Susannah O'Sullivan [a,*], Jian-Ming Lin [a], Maureen Watson [a], Karen Callon [a], Pak Cheung Tong [a], Dorit Naot [a], Anne Horne [a], Opetaia Aati [a], Fran Porteous [b], Greg Gamble [a], Jillian Cornish [a], Peter Browett [b], Andrew Grey [a]

[a] Department of Medicine, University of Auckland, Private Bag 92019, Auckland, New Zealand
[b] Department of Molecular Medicine and Pathology, University of Auckland, Private Bag 92019, Auckland, New Zealand

ARTICLE INFO

*Article history:*
Received 23 December 2010
Revised 13 April 2011
Accepted 17 April 2011
Available online 29 April 2011

Edited by: J. Aubin

*Keywords:*
Nilotinib
Osteoblasts
Mitogenesis
Platelet-derived growth factor
Osteoclasts
Osteoprotegerin

ABSTRACT

Nilotinib is a tyrosine kinase inhibitor (TKI) developed to manage imatinib-resistance in patients with chronic myeloid leukemia (CML). It inhibits similar molecular targets to imatinib, but is a significantly more potent inhibitor of Bcr-Abl. Nilotinib exhibits off-target effects in other tissues, and of relevance to bone metabolism, hypophosphataemia has been reported in up to 30% of patients receiving nilotinib. We have assessed the effects of nilotinib on bone cells *in vitro* and on bone metabolism in patients receiving nilotinib for treatment of CML. We firstly investigated the effects of nilotinib on proliferating and differentiating osteoblastic cells, and on osteoclastogenesis in murine bone marrow cultures and RAW264.7 cells. Nilotinib potently inhibited osteoblast proliferation (0.01–1 uM), through inhibition of the platelet-derived growth factor (PDGFR). There was a biphasic effect on osteoblast differentiation such that it was reduced by lower concentrations of nilotinib (0.1–0.5 uM), with no effect at higher concentrations (1 uM). Nilotinib also potently inhibited osteoclastogenesis, predominantly by stromal-cell dependent mechanisms. Thus, nilotinib decreased osteoclast development in murine bone marrow cultures, but did not affect osteoclastogenesis in RAW264.7 cells. Nilotinib treatment of osteoblastic cells increased expression and secretion of OPG and decreased expression of RANKL. In 10 patients receiving nilotinib, levels of bone turnover markers were in the low-normal range, despite secondary hyperparathyroidism, findings that are similar to those in patients treated with imatinib. Bone density tended to be higher than age and gender-matched normal values. These data suggest that nilotinib may have important effects on bone metabolism. Prospective studies should be conducted to determine the long-term effects of nilotinib on bone density and calcium metabolism.

© 2011 Elsevier Inc. All rights reserved.

## Introduction

Nilotinib is a tyrosine kinase inhibitor (TKI) developed to manage imatinib-resistance in patients with chronic myeloid leukemia (CML). Nilotinib inhibits similar molecular targets to imatinib, but is a significantly more potent inhibitor of Bcr-Abl than imatinib, with comparable potency in the inhibition of the platelet-derived growth factor (PDGFR) and c-KIT [1–4]. At therapeutic concentrations (1.7–3.6 μM) it may also inhibit the macrophage colony stimulating factor (M-CSF) receptor, and the collagen-related discoidin domain receptors (DDR-1 and −2) [5–7]. It has been used for the treatment of patients with CML who are resistant to or intolerant of other therapy [3,8–10], but may become a first-line agent because of recent evidence of greater efficacy than imatinib in treating CML [10]. Nilotinib is also being investigated for use in advanced gastrointestinal stromal tumors (GIST), systemic mastocytosis and hypereosinophilic syndrome [11–13]. As with other small molecule TKIs, nilotinib exhibits off-target or "bystander" effects due to inhibition of its molecular targets in healthy tissues [14]. Of relevance to bone metabolism, hypophosphataemia has been reported in up to 30% of patient receiving nilotinib for treatment of CML in Phase II and III clinical trials [10,15].

Studies published by our group and others have suggested that the related TKI, imatinib mesylate, has significant effects on bone and calcium metabolism [16–28]. *In vitro*, imatinib decreases osteoblast proliferation and survival, and increases osteoblast differentiation, actions which may be attributable to inhibition of PDGFR signaling [24,26–28]. Imatinib decreases osteoclastogenesis *in vitro*, by both direct and indirect, stromal-cell dependent mechanisms [19,20,24,29]. The similarities in the molecular targets of imatinib and nilotinib suggest that nilotinib might also affect skeletal cell function. Nilotinib has been reported to inhibit osteoclast formation and function, and promote

* Corresponding author. Fax: +64 9 3737677.
*E-mail addresses:* s.osullivan@auckland.ac.nz (S. O'Sullivan), j.lin@auckland.ac.nz (J.-M. Lin), m.watson@auckland.ac.nz (M. Watson), k.callon@auckland.ac.nz (K. Callon), pc.tong@auckland.ac.nz (P.C. Tong), d.naot@auckland.ac.nz (D. Naot), a.horne@auckland.ac.nz (A. Horne), o.aati@auckland.ac.nz (O. Aati), f.porteous@auckland.ac.nz (F. Porteous), g.gamble@auckland.ac.nz (G. Gamble), j.cornish@auckland.ac.nz (J. Cornish), p.browett@auckland.ac.nz (P. Browett), a.grey@auckland.ac.nz (A. Grey).

8756-3282/$ – see front matter © 2011 Elsevier Inc. All rights reserved.
doi:10.1016/j.bone.2011.04.014

osteoclast apoptosis [5], but to date there are no published data regarding its effects on osteoblasts or of its effects on bone and calcium metabolism *in vivo*. In the current work, we have assessed the actions of nilotinib on bone cells *in vitro* and in patients receiving nilotinib for treatment of CML.

## Materials and methods

### Cell culture

Primary rat osteoblastic cells were prepared as previously described [30]. The osteoblast-like character of these cells has been established by demonstration of high levels of alkaline phosphatase activity and osteocalcin production [31] and a sensitive adenylyl cyclase response to parathyroid hormone and prostaglandin E2 [32]. Murine pre-osteoblastic MC3T3-E1 cells (ATCC, Cryosite Distribution, Lane Cove, NSW, Australia), murine bone marrow stromal ST2 cells (St Vincent's Institute, Melbourne, Australia), and murine macrophage RAW264-7 cells (St Vincent's Institute, Melbourne, Australia) were maintained in standard cell culture conditions. Murine bone marrow cultures were established after harvesting marrow cells from the femora and tibiae of 4- to 6-week-old male animals, as previously described [33].

All protocols involving use of animals have been approved by the University of Auckland Animal Ethics Committee.

### Media and reagents

Minimum essential media (MEM), minimum essential media $\alpha$ modification ($\alpha$MEM), and Dulbecco's minimum essential media (DMEM) powder were purchased from Gibco BRL (Invitrogen Life Technologies, Carlsbad, CA). L-ascorbic acid-2-phosphate and $\beta$-glycerophosphate were purchased from Sigma-Aldrich Co. (St. Louis, MO). Imatinib mesylate and nilotinib were supplied by Novartis Pharma AG (Basel, Switzerland). Recombinant human osteoprotegerin (OPG) and murine receptor activator of nuclear factor $\kappa$B ligand (RANKL) were from Amgen Inc (Thousand Oaks, Ca). Rat platelet-derived growth factor (PDGF)-BB was from R&D Systems (Minneapolis, MN).

### Proliferation assay

Proliferation of osteoblastic cells was measured by cell counts and [$^3$H]-thymidine incorporation. In experiments designed to test the effects of nilotinib on proliferation, cells were cultured overnight in 5% FCS, then the media changed to 1% FCS at the time of addition of nilotinib. Cell numbers were analyzed 24 h after addition of nilotinib by detaching cells from the wells using trypsin/EDTA (0.05%/0.53 mM) for approximately 5 min at 37 C. Counting was performed in a hemocytometer chamber. [$^3$H]-thymidine incorporation was measured during a 6 h window at the end of 24 h of treatment, as previously described [30,34]. In experiments designed to test the effects of nilotinib on PDGF-stimulated osteoblast mitogenesis, cells were cultured overnight in 5% FCS, then placed in serum-free media for 24 h prior to addition of PDGF with or without nilotinib for 24 h, with measurement of [$^3$H]-thymidine incorporation during a 6 h window at the conclusion of the treatment period. In these experiments, nilotinib-only controls were included and [$^3$H]-thymidine incorporation induced by PDGF in the presence or absence of nilotinib was expressed as a ratio to the values obtained in cultures treated with vehicle or nilotinib alone, respectively.

### Live/dead cell viability assay

Cell viability was assessed in primary rat osteoblasts treated with vehicle or nilotinib using a Live/Dead Viability/Cytotoxicity Kit (Invitrogen), according to the manufacturer's instructions.

### Mineralization assay

MC3T3-E1 cells were plated in 6-well tissue culture dishes, at a density of $5\times10^4$ cells/well, in 10% FCS/MEM/sodium pyruvate. When cells were confluent (approximately 3 days after plating) and no longer proliferating, media were changed to 15% FCS/$\alpha$MEM supplemented with 50 μg/ml L-ascorbic acid-2-phosphate and 10 mM $\beta$-glycerophosphate, and test substances were added. These supplemented media were changed twice weekly and test substances were replaced. After 18–21 days, the cells were fixed in neutral buffered formalin, rinsed thoroughly with distilled water, and the cultures stained for mineral using Von Kossa stain. Quantification of mineralized areas was performed using a semi-automatic image analysis Bioquant system (Bioquant Image Analysis Corporation, Nashville, TN).

### Osteoclastogenesis assays

Assays of osteoclastogenesis in RAW264.7 cells and murine bone marrow cells were performed as previously described [24]. After culture for 4–5 day (RAW264.7 cells) or 7 day (bone marrow), cells were fixed and stained for tartrate-resistant acid phosphatase (TRAP). Multinucleated cells (containing three or more nuclei) positive for TRAP were counted. In addition to the previously described cultures, murine bone marrow cultures were performed with nilotinib addition at a later time point in the experiment.

### Osteoprotegerin assay

Osteoprotegerin, an endogenous inhibitor of RANKL signaling, was measured in conditioned media from cultures of cells treated with vehicle or nilotinib, using the murine osteoprotegerin/TNFRSF11B DuoSet (R&D Systems, Minneapolis, MN), according to the manufacturer's instructions.

### Analysis of gene expression

Total cellular RNA was extracted from cultured cells and purified using RNeasy minikit (Qiagen). Genomic DNA was removed using RNase-free DNase set (Qiagen). Reverse transcription was carried out as previously described [35], and cDNA was used for real-time PCR. The primer-probe sets were purchased from Applied Biosystems (Foster City, CA). Multiplex PCR was performed with FAM-labeled probes specific for the gene of interest, and VIC-labeled 18S rRNA probes according to the company's instructions, using ABI PRISM 7900HT sequence detection system (Applied Biosystems). Samples were assayed in duplicate or triplicate. The relative level of mRNA expression was determined using the $\Delta\Delta$Ct calculation method as previously described [35]. Expression data were normalized to the control value at the earliest time point assayed.

### RNA interference

RNA interference ("gene-silencing") was performed using Stealth select probes specific murine *PDGFRB* (Invitrogen catalogue # MSS207504), *PDGFRA* (Invitrogen catalogue #MSS207501) or for murine *ABL-1* (Invitrogen catalogue # MSS235749) or a GC control sequence. ST2 cells were seeded into 24 well plates at a density of $5\times10^4$ cells/ml in DMEM/5% FCS and incubated for 24 h. Cells were then transfected with the *PDGFRB, PDGFRA* or the *ABL-1* probe or the GC control probe using Lipofectamine transfection reagent. After 6 h, media was changed to DMEM/1% FCS and the plates were treated as for a standard proliferation assay as described above. In cultures treated in parallel, RNA was collected after 24 h for analysis of expression of the target gene of interest.

### Subjects

The study population consisted of ten subjects with BCR/ABL positive CML who had been treated with nilotinib for a median of

**Table 1**
Demographic, disease and treatment characteristics of study subjects.

| Variable | Value |
|---|---|
| Age (y) | 49 [32–56] |
| Gender (M/F) | 3/7 |
| Menopausal Status (pre/post) | 4/3 |
| Body mass index (kg/m$^2$) | 27 [19–41] |
| Disease duration (months) | 72 [32–159] |
| Number with cytogenetic response | 9 |
| Duration of nilotinib treatment (months) | 32 [12–54] |
| Number taking nilotinib 800 mg/day | 9[a] |

Data are *n*, or median [range].
[a] Nilotinib dose was 400 mg daily in one subject (see text).

32 months (range 12–54). The demographic, disease and treatment data are shown in Table 1. None had metabolic bone disease or impaired renal function, but two patients were receiving calcium supplements, one of whom had had low-normal serum calcium (8.2 mg/dL) while taking imatinib. Seven subjects had received 400 mg of nilotinib twice daily since starting treatment. Nilotinib dose was reduced to 400 mg daily in three subjects because of drug-related side-effects (pancytopenia, headache and malaise); in two cases the dose was increased back to 400 mg twice daily within four months, while the third patient remained on 400 mg daily. At the time of sampling, all but one patient was taking 800 mg daily and had attained a complete cytogenetic response. All ten patients had previously received imatinib, for a median duration of 32 months (range 9–68). Imatinib therapy was stopped and nilotinib started because of failure to achieve a major cytogenetic response in six subjects, and loss of cytogenetic response in four patients.

Previously published data from a prospectively studied cohort of patients with CML treated with imatinib for 2 years, in which we reported the development of secondary hyperparathyroidism and decreased bone turnover, [23], are included here for comparative purposes. None of the nilotinib-treated patients had participated in the imatinib study.

The study was approved by the Northern X Regional Ethics Committee (approval number NTX/09/03/022), and all participants gave written informed consent.

*Biochemistry*

All subjects provided serum, plasma and urine samples, after overnight fast. Serum calcium, albumin, phosphate and creatinine, and urine phosphate and creatinine were measured on a Roche Modular autoanalyser. Albumin-adjusted serum calcium was calculated using the formula sCa adj = total sCa–0.8 (sAlbumin [g/dL]–4). Tubular maximum for phosphate reabsorption (TmP/GFR) was calculated as previously described [36]. Serum 25(OH)D was measured by radioimmunoassay (DiaSorin, Stillwater, Mn). Intact parathyroid hormone (PTH) and total testosterone were measured using electrochemiluminescence immunoassays (E170, Roche, Basel, Switzerland).

*Bone turnover markers*

Serum levels of β-C-terminal telopeptide of type I collagen (βCTX) and procollagen type-I N-terminal propeptide (PINP) were measured

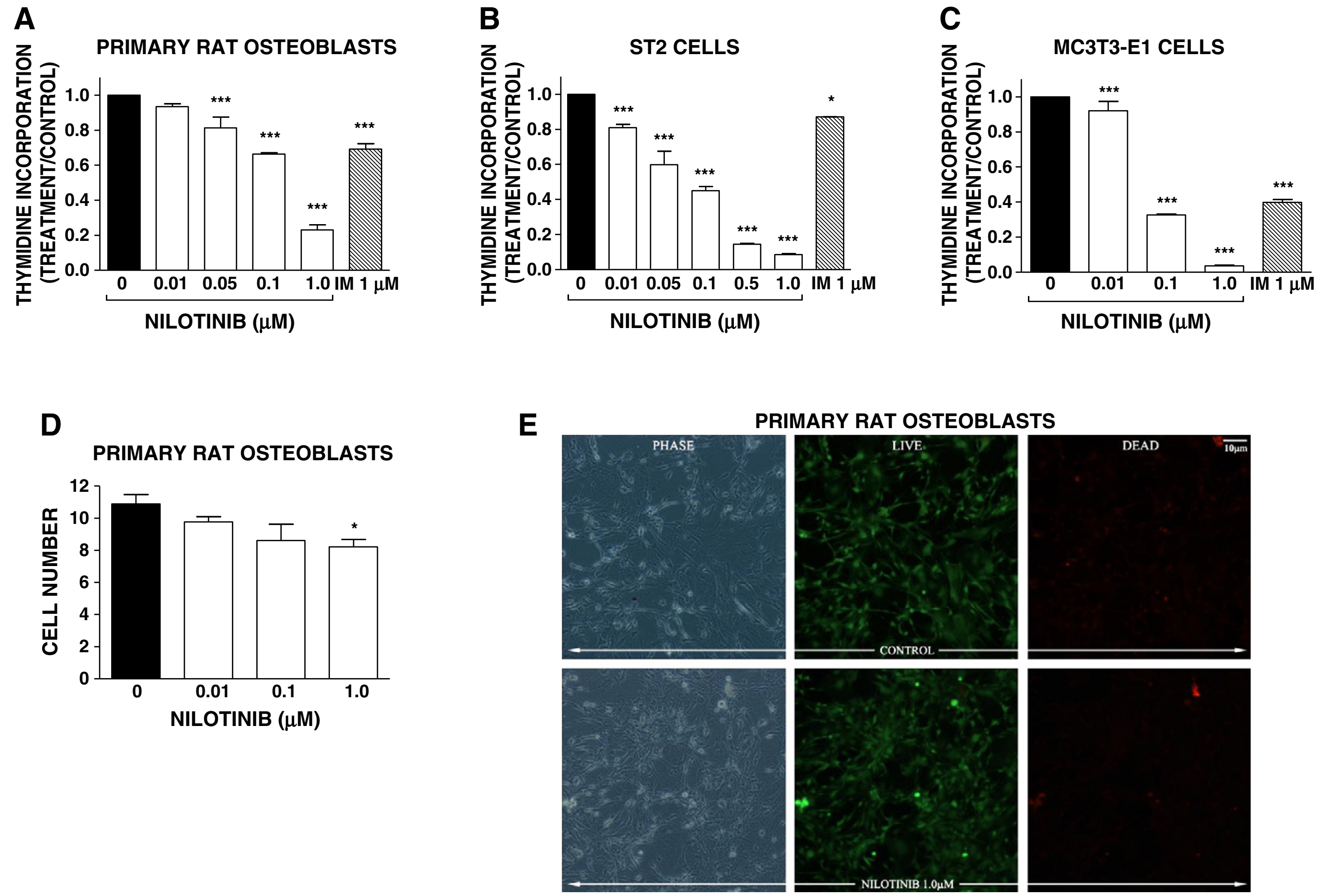


**Fig. 1.** Effects of nilotinib on [$^3$H]-thymidine incorporation in (A) primary rat osteoblasts, (B) ST2 stromal cells, and (C) MC3T3-E1 cells. (D) Effects of nilotinib on cell number in primary rat osteoblasts. (E) Representative image of effects of nilotinib on cell viability in primary rat osteoblasts. Data are mean ± SEM. *, $p<0.05$ vs control; ***, $p<0.001$ vs control. IM, imatinib.

as previously described [37]. Coefficients of variation of these markers are as follows: PINP, 1.9%; βCTX, 5.1%.

*Bone mineral density*

BMD was measured using a Lunar Prodigy densitometer (GE Lunar, Madison, WI), as previously described [23]. Body weight was measured using electronic scales.

*Statistical analyses*

Data were analyzed using GraphPad Prism (v5.04) (GraphPad Software, San Diego, CA). Data from experiments evaluating multiple time points or drug/peptide concentrations were analyzed by repeated measures analysis of variance (ANOVA), with Dunnett's post-hoc test. Paired data were analyzed by Student's *t* test. Data from nilotinib-treated patients were compared with the midpoint of the laboratory normal range using Student's related groups test, or the Wilcoxon signed rank test if data were not normally distributed. Data from nilotinib-treated patients were compared with imatinib-treated patients using Student's *t* test.

## Results

*Nilotinib is a potent inhibitor of osteoblast proliferation and this effect is mediated by the PDGFR*

At concentrations comparable to or lower than those that occur in serum in patients treated with standard doses of nilotinib [14], there was a dose-dependent inhibition of proliferation of actively growing primary rat osteoblastic cells as evidenced by reduced [$^3$H]-thymidine incorporation and a reduction in cell number (Figs. 1A and D respectively). At the same concentrations, nilotinib inhibited mitogenesis of ST2 cells and MC3T3-E1 cells (Figs. 1B and C). This was not a non-specific cytotoxic effect of nilotinib, as there was no evidence of cell detachment or increased death in the primary rat osteoblasts (Figs. 1E). When compared to imatinib, nilotinib more potently inhibited mitogenesis in each of the osteoblast cultures. In previous work we have shown that the effects of imatinib mesylate on osteoblast proliferation are mediated by the PDGFR [24]. We therefore investigated whether inhibition of PDGFR signaling also mediates the anti-proliferative effects of nilotinib. In primary rat osteoblasts, ST2 cells and MC3T3-E1 cells, PDGF potently stimulated mitogenesis of osteoblastic cells, an effect that was reversed by co-treatment with nilotinib (Figs. 2A–C). This was not a non-specific inhibitory effect, as co-treatment with nilotinib did not inhibit the mitogenic effect of lactoferrin (Fig. 2C). To confirm these findings in a more direct fashion, we used gene silencing techniques to inhibit expression of the *PDGFRB* and *PDGFRA* genes. Using these techniques, *PDGFRB* gene expression was reduced by more than 70% leading to a 40% inhibition of mitogenesis in ST2 cells (Fig. 3A). A similar degree of inhibition of *PDGFRA* did not significantly reduce mitogenesis (Fig. 3B). We also considered the possibility that inhibition by nilotinib of one of its other targets may be contributing to the effects we observed. However, reducing *ABL* gene expression by more than 70% did not significantly affect mitogenesis of ST2 cells (Fig. 3C). As ST2 cells do not express the *KIT* gene [24], we did not further investigate a role for c-KIT in mediating the anti-proliferative effects of nilotinib.

*Nilotinib inhibits differentiation of osteoblastic cells*

In contrast to imatinib, nilotinib had an inhibitory effect on the formation of mineralized tissue in long-term cultures of osteoblastic cells. The effect of nilotinib appeared to be biphasic, such that it

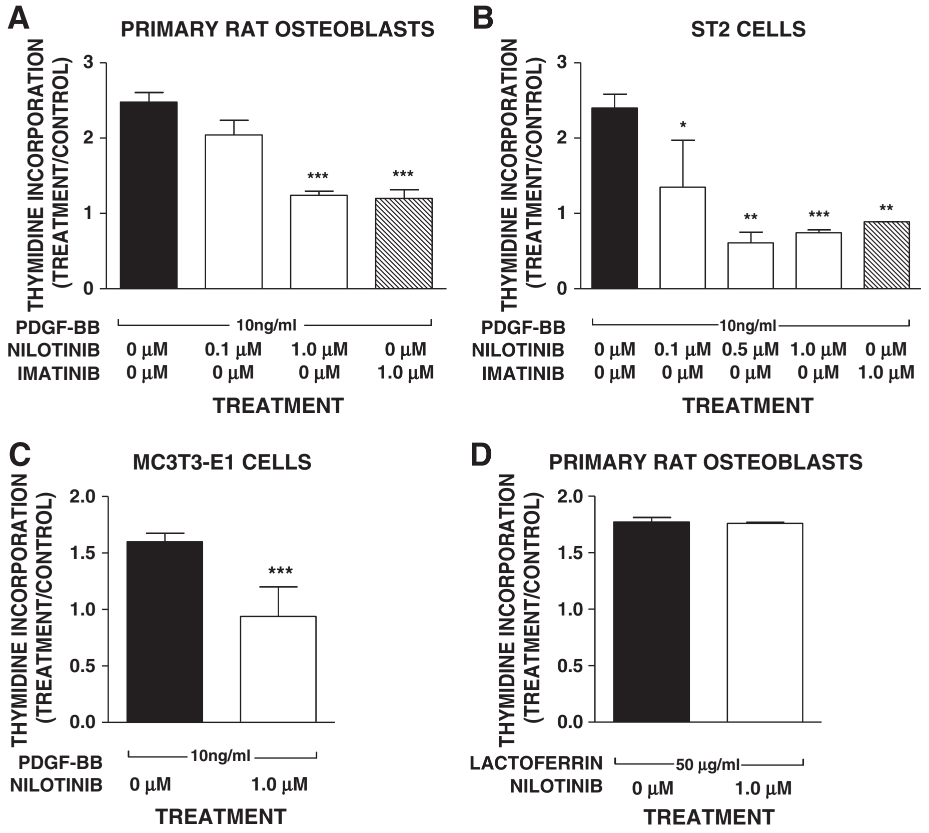


**Fig. 2.** The effects of nilotinib on osteoblast mitogenesis are mediated by inhibition of PDGFR signaling. PDGF stimulates mitogenesis of (A) primary rat osteoblasts, (B) ST2 cells, and (C) MC3T3-E1 cells, effects that are inhibited by nilotinib in each cell type. (D) Nilotinib does not inhibit lactoferrin-induced osteoblast mitogenesis. Data are mean ± SEM. *, $p<0.05$ vs PDGF; **, $p<0.01$ vs PDGF; ***, $p<0.001$ vs PDGF.

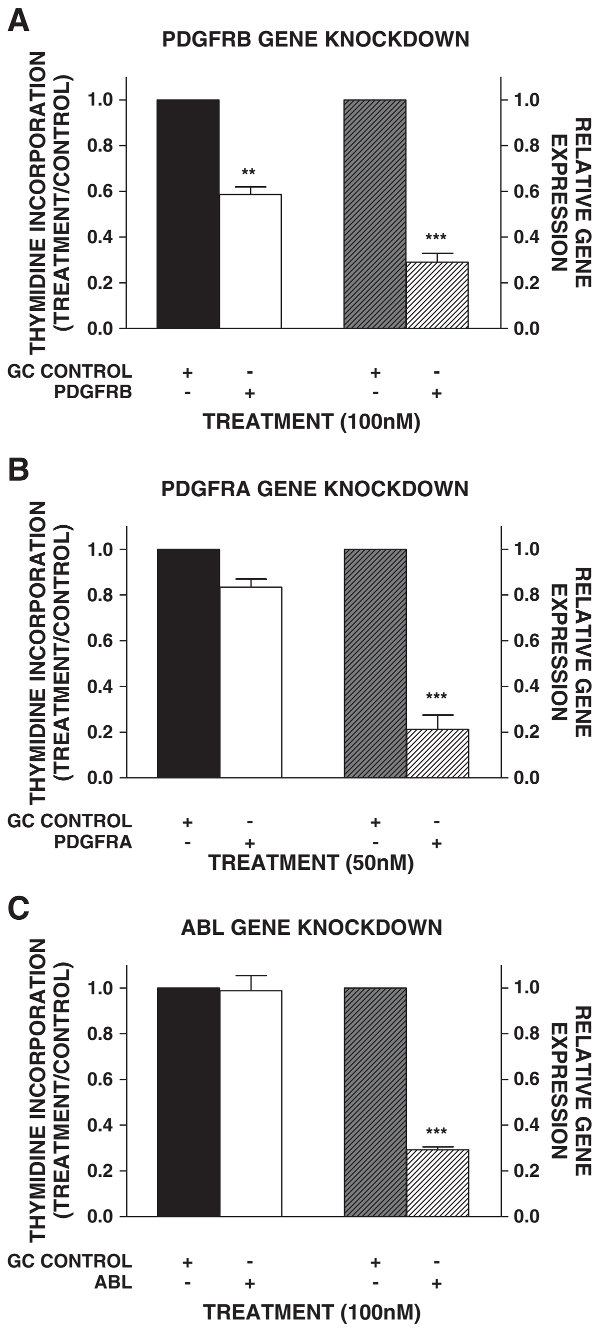


**Fig. 3.** PDGFRβ signals osteoblast mitogenesis. Effects of siRNA targeting (A) PDGFRB, (B) PDGFRA or (C) ABL on [$^3$H]-thymidine incorporation in ST2 cells. Cells are treated with siRNA for the gene of interest or GC control sequence. In each panel, bars on left show [$^3$H]-thymidine incorporation, bars on right show gene expression, determined by q-PCR. Data are mean ± SEM. **, $p<0.01$ vs control oligo; ***, $p<0.001$ vs control oligo.

inhibited differentiation of MC3T3-E1 cells at concentrations of 0.1 μM and 0.5 μM, while having a neutral effect at 1.0 μM (Figs. 4A and B).

*Nilotinib inhibits osteoclastogenesis*

We assessed the effects of nilotinib on osteoclast development in vitro, using murine bone marrow cultures stimulated with 1,25 $(OH)_2D$. The number of newly developed osteoclasts was significantly decreased by nilotinib at concentrations of 0.5 μM and higher (Figs. 5A and B). In a similar fashion to imatinib, there appeared to be a dose-dependent reduction in the number of stromal cells in the presence of nilotinib, consistent with the anti-proliferative effects we observed in osteoblastic cells. When nilotinib was added at a later time-point the inhibitory effect on osteoclastogenesis was less marked than when it was present for all of the experimental period (Fig. 5C). Stromal cell numbers were reduced in both cases, but to a lesser extent when nilotinib was added later in the assay. To further investigate whether the inhibition of osteoclastogenesis by nilotinib was attributable to indirect actions on the stromal cell population, we assessed the effect of nilotinib on formation of osteoclasts using the RAW-264.7 macrophage cell line. In these cultures, stromal cells are not present and TRAP-positive multinucleated osteoclasts develop in response to treatment with RANKL. Nilotinib did not inhibit the development of osteoclasts in RANKL-treated RAW-264.7 cells (Fig. 5D).

A mechanism by which nilotinib might indirectly inhibit osteoclastogenesis is by altering stromal cell production of cytokines known to influence osteoclast development [38]. *RANKL* mRNA expression was reduced in cultures of ST2 cells (data not shown), and *OPG* mRNA expression was increased in cultures of ST2 cells and murine bone marrow culture treated with nilotinib (Figs. 5E and F). We measured OPG protein in cell-conditioned medium from ST2 cells treated with nilotinib and found that it was significantly increased over control values (Fig. 5G). These data suggest that an increase in OPG production by stromal/ osteoblastic cells may contribute to the anti-resorptive actions of nilotinib, in addition to its effect to reduce stromal cell number. Taken together, these data suggest that nilotinib may inhibit osteoclastogenesis by a stromal cell-dependent mechanism(s), however, we cannot exclude the possibility that nilotinib may have a direct inhibitory effect on the development of osteoclast precursors, or on survival of pre-osteoclasts.

*Patients treated with nilotinib have secondary hyperparathyroidism and low-normal markers of bone turnover*

*Biochemistry*

The patients treated with nilotinib had secondary hyperparathyroidism. Mean levels of calcium and phosphate were at the lower end of the normal range (mean [SD] 8.5 [0.3] mg/dL and 2.9 [0.6] mg/dL respectively, $p<0.001$ and $p<0.05$ vs normal population mean) (Figs. 6A and B), and the mean level of PTH was at the upper end of the normal range (mean [SD] 59.9 [40.2] pg/ml, $p<0.05$ vs normal population mean) (Fig. 6D). One patient had a PTH level above the upper limit of the normal range. The mean level of TmP was below the normal range (mean [SD] 2.5 [0.7] mg/dL GFR, $p<0.01$ vs normal population mean) (Fig. 6C). Levels of each of these biochemical parameters were similar to those in patients with CML treated with imatinib for 2 years [23] which are reproduced here for comparative purposes (Figs. 6A–D). In the nilotinib-treated patients, mean (SD) estimated glomerular filtration rate was 106 (20) ml/min/1.73 $m^2$, and serum 25OHD was 35 (15) nmol/L.

*Bone turnover*

In patients treated with nilotinib, mean levels of the bone formation marker P1NP and the bone resorption marker, βCTX, were in the lower part of the normal range, and each was significantly different to the mean value in healthy premenopausal women (mean [SD] 28.9 [12.4] μg/L and 149 [97] ng/L respectively, $p=0.0004$ and $p=0.0008$ respectively) (Figs. 6E and F). Both values were higher than in imatinib-treated patients (mean [SD] 15.6 [3.1] μg/L, $p=0.025$ vs nilotinib-treated patients and 90 [31] ng/L, $p=0.02$ vs nilotinib-treated patients respectively). The patient receiving a low dose of nilotinib (400 mg daily) had a P1NP level above the middle of the normal premenopausal range. Two nilotinib-treated post-menopausal patients had βCTX levels at the mid-point of the normal premenopausal range.

*Bone mineral density*

The mean bone mineral density (BMD) in the nilotinib-treated patients tended to be higher than the age and gender-matched normal mean value at the total body and lumbar spine sites but not at the

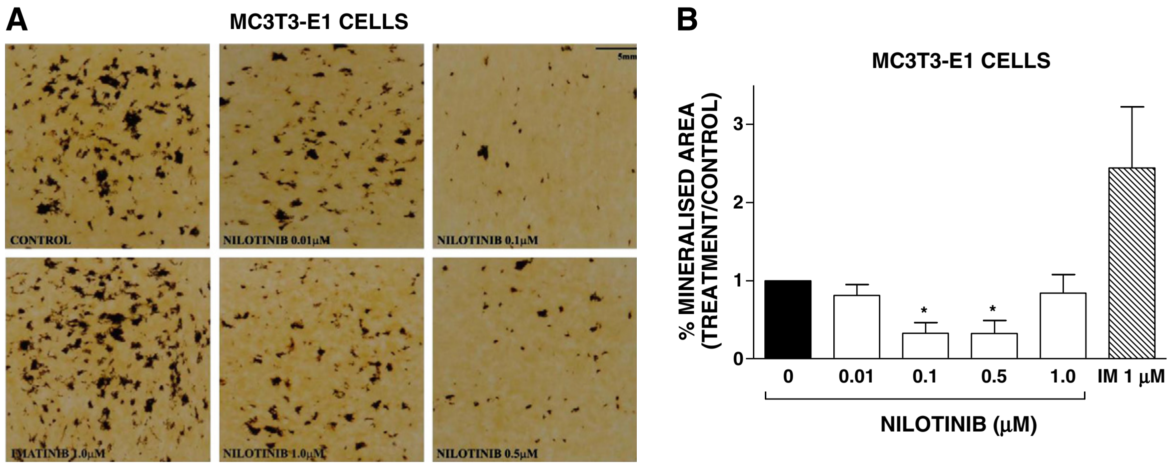


**Fig. 4.** Effects of nilotinib on differentiation of MC3T3-E1 cells. (A) Representative images of mineralized nodules (black, von Kossa stain) in MC3T3-E1 cells treated with the indicated concentrations of nilotinib or imatinib. (B) Percentage mineralised area in cultures of cells treated with the indicated concentrations of nilotinib or imatinib. Data are mean ± SEM. *, $p<0.05$ vs control; ***, $p<0.001$ vs control.

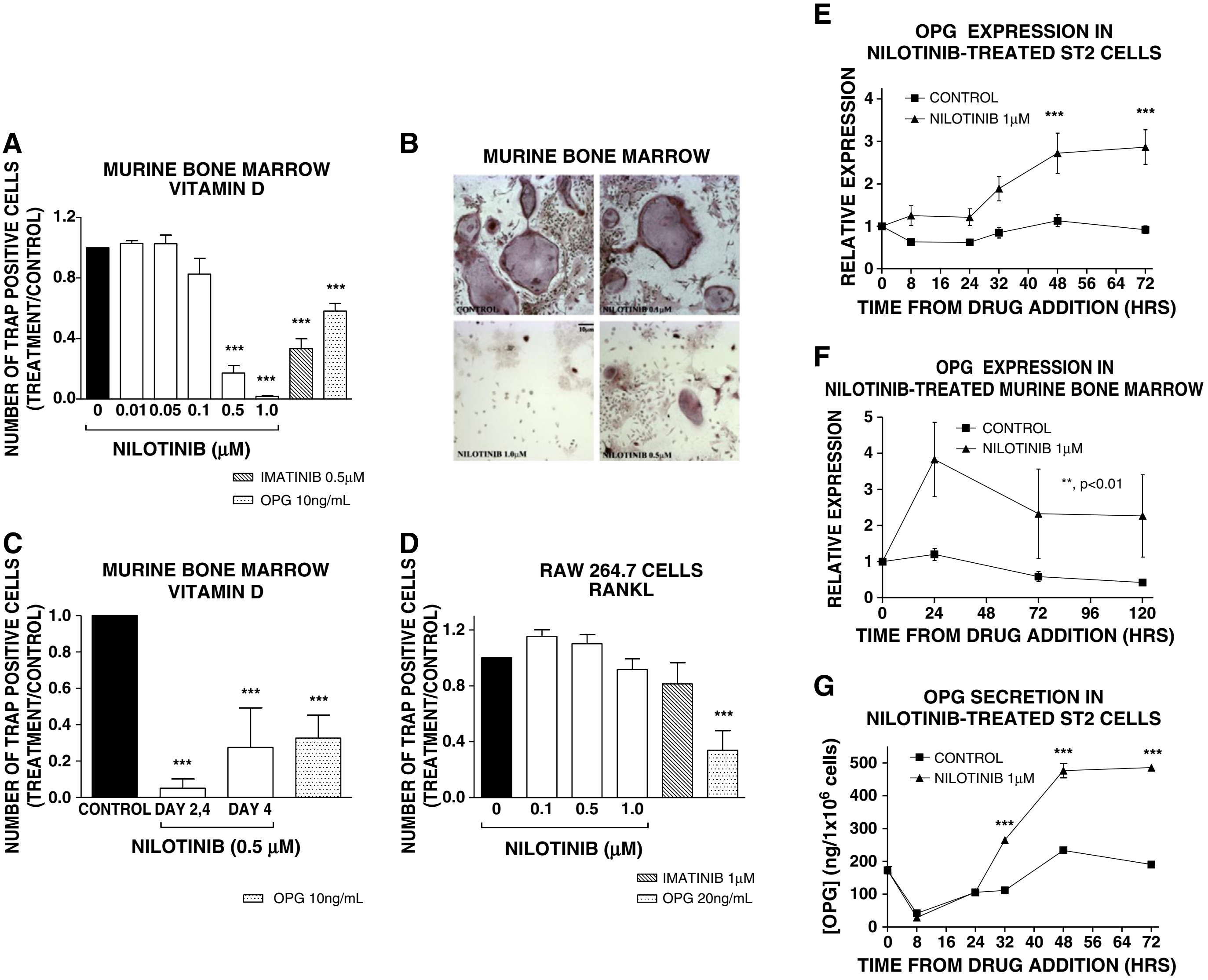


**Fig. 5.** (A) Effects of nilotinib on osteoclastogenesis in murine bone marrow cultures treated with 1,25(OH)$D_3$. (B) Representative images of osteoclasts in murine bone marrow cultures treated with 1,25(OH)$D_3$ and the indicated concentrations of nilotinib. (C) Effects of nilotinib on osteoclastogenesis in murine bone marrow cultures treated with 1,25(OH) $D_3$ when nilotinib is added at a later time-point in the assay. (D) Effects of nilotinib on osteoclastogenesis in RAW-264.7 cells treated with RANKL 10 ng/ml. Data are mean ± SEM ***, $p<0.001$ vs control. Effect of nilotinib on expression of *OPG* mRNA in (E) ST2 cells and (F) murine bone marrow. Gene expression is quantitated relative to the baseline value. (G) Effect of nilotinib on production of OPG protein by ST2 cells. Data are mean ± SEM. *, $p<0.05$ vs vehicle; ***, $p<0.001$ vs vehicle. OPG, osteoprotegerin.

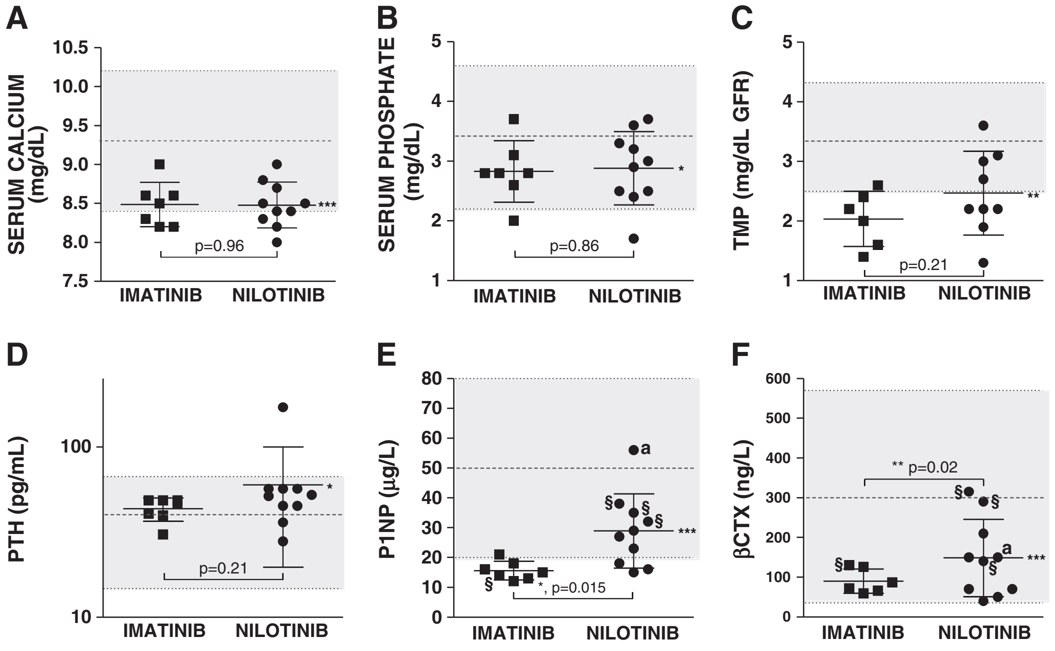

**Fig. 6.** (A–D) Biochemical measurements in CML patients treated with nilotinib or imatinib. Data from imatinib-treated patients have previously been published [23] and are reproduced here for comparative purposes. A, albumin-adjusted serum calcium; B, serum phosphate; C, tubular maximum for phosphate reabsorption (TmP/GFR); D, intact parathyroid hormone (PTH) (Note — data have been logarithmically transformed as the nilotinib data are not normally distributed); (E–F) Levels of bone turnover markers in CML patients treated with nilotinib. E, serum procollagen type-I N-terminal propeptide (P1NP); F, serum β-C-terminal telopeptide of type I collagen (βCTX). The shaded areas in A–D represent the laboratory normal ranges, and in E–F represent the normal ranges for healthy premenopausal women. Interrupted lines indicate the mean value in the reference population. Horizontal bars indicate mean and SD. *, $p<0.05$ vs mean of the normal range; **, $p<0.01$ vs mean of the normal range; ***, $p<0.001$ vs mean of the normal range. a, patient taking low dose of nilotinib (400 mg/day); §, postmenopausal women. GFR, glomerular filtration rate. To convert to SI units: calcium, divide by 4; phosphate, divide by 3.1; TmP, divide by 3.1; PTH, divide by 9.

proximal femur (z-score mean [SD] total body 0.99 [1.30], $p=0.04$; lumbar spine 0.69 [1.01], $p=0.06$; total hip 0.60 [1.30], $p=0.18$). There were no significant differences between the BMD of the nilotinib treated patients and the patients treated with imatinib for 2 years (data not shown).

## Discussion

Small molecule TKIs are emerging as powerful tools in the management of an array of diseases. Imatinib mesylate is the prototype TKI, and studies published by our group and others suggest that it has important effects on bone and calcium metabolism [16–28]. Nilotinib is active against a similar array of molecular targets to imatinib but is more potently inhibitory of the ABL tyrosine kinase. Recent evidence that nilotinib is superior to imatinib in achieving remission in CML makes it likely that nilotinib will be more frequently prescribed in the future [10]. In this work we found that nilotinib, at concentrations similar to those observed *in vivo* [14] inhibits osteoblast proliferation without affecting cell viability, has a neutral or inhibitory effect on osteoblast differentiation, and inhibits osteoclastogenesis in vitro. In patients, nilotinib decreases bone turnover, despite causing secondary hyperparathyroidism.

As with imatinib [24], nilotinib's inhibitory effect on osteoblast proliferation is mediated through PDGFR signaling. Thus, in each of three osteoblastic cell types, nilotinib inhibited the mitogenic effect of PDGF. Gene silencing demonstrated that the PDGFRβ is most likely to be the critical receptor in mediating these effects since siRNA targeting of this receptor decreases osteoblast mitogenesis. Gene silencing of *PDGFRA* and *ABL* had no effect on osteoblast mitogenesis, and osteoblasts from $ABL^{-/-}$mice proliferate normally [39], arguing against a pivotal role for either PDGFRα or c-ABL in mediating nilotinib's inhibitory effects on osteoblast proliferation. In *contrast* to imatinib, which induces osteoblast differentiation at concentrations greater than 0.05 μM [24], nilotinib inhibits or has a neutral effect on osteoblast differentiation. It has previously been reported that $ABL^{-/-}$mice have dysfunctional osteoblasts, with impaired differentiation, while we and others have reported that the stimulatory effects of imatinib on differentiation of osteoblasts may be mediated by inhibition of the PDGFR [24,27,28]. Thus, we speculate that the effects of nilotinib on osteoblast differentiation result from the relative balance of its inhibitory effects on c-ABL and the PDGFR. At doses <1.0 μM the c-ABL inhibitory effect predominates, leading to inhibition of differentiation. At higher doses there is a significant PDGFR inhibitory effect, which rescues the impairment of osteoblast differentiation induced by inhibition of c-ABL.

The current work also demonstrates that nilotinib inhibits osteoclastogenesis, in keeping with the findings of a previous study [5] and with previous findings with imatinib [19,20,24]. In a similar fashion to that seen with imatinib, we found that nilotinib potently inhibited osteoclastogenesis in the murine bone marrow assay treated with 1,25 $(OH)_2D$, in which stromal cell-derived osteoclastogenic cytokines play a critical role in driving maturation of osteoclast precursors. Experiments assessing the direct effect of nilotinib on osteoclast development, performed by using the stromal cell-independent RAW-264.7 cells, suggested that the anti-osteoclastogenic actions of nilotinib are largely stromal cell-dependent, however the possibility that nilotinib has a direct inhibitory effect on osteoclast precursors cannot be excluded. Our findings suggest that nilotinib induces stromal cell-dependent inhibition of osteoclastogenesis by limiting the number of stromal cells available to support differentiation of osteoclast precursors and by increasing the production by stromal cells of osteoprotegerin, a secreted decoy receptor for RANKL. The findings that both imatinib [24] and nilotinib (current work) increase stromal cell production of OPG suggest that signaling via a common molecular target regulates the synthesis of this critical endogenous regulator of osteoclastogenesis.

We have demonstrated that patients receiving nilotinib for treatment of CML have alterations in bone and calcium metabolism that are similar in nature and magnitude to those observed in patients treated with imatinib [23]. Thus, patients treated with nilotinib have

decreased bone turnover, despite secondary hyperparathyroidism. The reason(s) for the effects of TKIs on calcium and phosphate metabolism remain uncertain. In imatinib-treated patients, secondary hyperparathyroidism persists for at least 2 years, well beyond the time during which uncoupling of bone formation and bone resorption is observed [23], and the mean duration of nilotinib therapy in the current study is more than 2 years. Thus, a non-skeletal action, such as decreased intestinal calcium absorption, may explain the persistent secondary hyperparathyroidism in patients receiving imatinib or nilotinib. The current study is limited by its cross-sectional design and by the fact that each patient had previously received imatinib for at least 9 months and most for more than 2 years. It is not known whether the effects of imatinib on calcium metabolism are reversible, and although it seems unlikely, we cannot exclude the possibility that the effects we observed in the nilotinib-treated patients are the result of irreversible actions of the imatinib therapy each patient had previously received.

The bone turnover data are in keeping with the *in vitro* data that demonstrate that nilotinib inhibits both osteoblast differentiation and osteoclastogenesis, while also inhibiting proliferation of immature osteoblastic cells. Bone density tended to be higher than normal in the nilotinib-treated group, raising the possibility that it exerts an anti-resorptive effect *in vivo*, but the cross-sectional nature of this study means that it is not possible to definitively determine whether nilotinib alters bone density. Similarly we were not able to determine whether patients taking nilotinib experienced the significant gains in weight and fat mass that we observed in patients treated with imatinib [23].

In summary, nilotinib inhibits osteoblast proliferation and differentiation, and osteoclastogenesis *in vitro*, and leads to low levels of bone turnover and secondary hyperparathyroidism in patients being treated for CML. Cross-sectional bone density data suggest that nilotinib therapy might increase bone density. Prospective studies of the effects of nilotinib on bone and calcium metabolism should be undertaken.

## Conflict of interest

Peter Browett has received research funding and consulting fees from Novartis. All other authors have nothing to declare.

## Acknowledgments

This work was funded by the Health Research Council of New Zealand and the Auckland Medical Research Foundation. Dr. O'Sullivan is the recipient of a Postdoctoral Scholarship from the Auckland Medical Research Foundation and an Emerging Researcher grant from the Health Research Council of New Zealand.

## References

[1] Weisberg E, Manley P, Mestan J, Cowan-Jacob S, Ray A, Griffin JD. AMN107 (nilotinib): a novel and selective inhibitor of BCR-ABL. Br J Cancer 2006;94: 1765–9.
[2] Verstovsek S, Akin C, Manshouri T, Quintas-Cardama A, Huynh L, Manley P, et al. Effects of AMN107, a novel aminopyrimidine tyrosine kinase inhibitor, on human mast cells bearing wild-type or mutated codon 816 c-kit. Leuk Res 2006;30: 1365–70.
[3] Weisberg E, Manley PW, Breitenstein W, Bruggen J, Cowan-Jacob SW, Ray A, et al. Characterization of AMN107, a selective inhibitor of native and mutant Bcr-Abl. Cancer Cell 2005;7:129–41.
[4] Manley PW, Cowan-Jacob SW, Mestan J. Advances in the structural biology, design and clinical development of Bcr-Abl kinase inhibitors for the treatment of chronic myeloid leukaemia. Biochim Biophys Acta 2005;1754:3–13.
[5] Brownlow N, Russell AE, Saravanapavan H, Wiesmann M, Murray JM, Manley PW, et al. Comparison of nilotinib and imatinib inhibition of FMS receptor signaling, macrophage production and osteoclastogenesis. Leukemia 2008;22:649–52.
[6] Taylor JR, Brownlow N, Domin J, Dibb NJ. FMS receptor for M-CSF (CSF-1) is sensitive to the kinase inhibitor imatinib and mutation of Asp-802 to Val confers resistance. Oncogene 2006;25:147–51.
[7] Manley PW, Drueckes P, Fendrich G, Furet P, Liebetanz J, Martiny-Baron G, et al. Extended kinase profile and properties of the protein kinase inhibitor nilotinib. Biochim Biophys Acta 2010;1804:445–53.
[8] Shah NP, Tran C, Lee FY, Chen P, Norris D, Sawyers CL. Overriding imatinib resistance with a novel ABL kinase inhibitor. Science 2004;305:399–401.
[9] Ray A, Cowan-Jacob SW, Manley PW, Mestan J, Griffin JD. Identification of BCR-ABL point mutations conferring resistance to the Abl kinase inhibitor AMN107 (nilotinib) by a random mutagenesis study. Blood 2007;109:5011–5.
[10] Saglio G, Kim DW, Issaragrisil S, le Coutre P, Etienne G, Lobo C, et al. Nilotinib versus imatinib for newly diagnosed chronic myeloid leukemia. N Engl J Med 2010;362:2251–9.
[11] Montemurro M, Schoffski P, Reichardt P, Gelderblom H, Schutte J, Hartmann JT, et al. Nilotinib in the treatment of advanced gastrointestinal stromal tumours resistant to both imatinib and sunitinib. Eur J Cancer 2009;45:2293–7.
[12] von Bubnoff N, Gorantla SP, Thone S, Peschel C, Duyster J. The FIP1L1-PDGFRA T674I mutation can be inhibited by the tyrosine kinase inhibitor AMN107 (nilotinib). Blood 2006;107:4970–1.
[13] von Bubnoff N, Gorantla SH, Kancha RK, Lordick F, Peschel C, Duyster J. The systemic mastocytosis-specific activating cKit mutation D816V can be inhibited by the tyrosine kinase inhibitor AMN107. Leukemia 2005;19:1670–1.
[14] Kantarjian H, Giles F, Wunderle L, Bhalla K, O'Brien S, Wassmann B, et al. Nilotinib in imatinib-resistant CML and Philadelphia chromosome-positive ALL. N Engl J Med 2006;354:2542–51.
[15] Kantarjian HM, Giles FJ, Bhalla KN, Pinilla-Ibarz J, Larson RA, Gattermann N, et al. Nilotinib is effective in patients with chronic myeloid leukemia in chronic phase after imatinib resistance or intolerance: 24-month follow-up results. Blood 2011;117:1141–5.
[16] Berman E, Nicolaides M, Maki RG, Fleisher M, Chanel S, Scheu K, et al. Altered bone and mineral metabolism in patients receiving imatinib mesylate. N Engl J Med 2006;354:2006–13.
[17] Dewar AL, Doherty KV, Hughes TP, Lyons AB. Imatinib inhibits the functional capacity of cultured human monocytes. Immunol Cell Biol 2005;83:48–56.
[18] Dewar AL, Domaschenz RM, Doherty KV, Hughes TP, Lyons AB. Imatinib inhibits the in vitro development of the monocyte/macrophage lineage from normal human bone marrow progenitors. Leukemia 2003;17:1713–21.
[19] Dewar AL, Farrugia AN, Condina MR, Bik To L, Hughes TP, Vernon-Roberts B, et al. Imatinib as a potential antiresorptive therapy for bone disease. Blood 2006;107: 4334–7.
[20] El Hajj Dib I, Gallet M, Mentaverri R, Sevenet N, Brazier M, Kamel S. Imatinib mesylate (Gleevec) enhances mature osteoclast apoptosis and suppresses osteoclast bone resorbing activity. Eur J Pharmacol 2006;551:27–33.
[21] Grey A, O'Sullivan S, Reid IR, Browett P. Imatinib mesylate, increased bone formation, and secondary hyperparathyroidism. N Engl J Med 2006;355:2494–5.
[22] Jonsson S, Olsson B, Ohlsson C, Lorentzon M, Mellstrom D, Wadenvik H. Increased cortical bone mineralization in imatinib treated patients with chronic myelogenous leukemia. Haematologica 2008;93:1101–3.
[23] O'Sullivan S, Horne A, Wattie D, Porteous F, Callon K, Gamble G, et al. Decreased bone turnover despite persistent secondary hyperparathyroidism during prolonged treatment with imatinib. J Clin Endocrinol Metab 2009;94:1131–6.
[24] O'Sullivan S, Naot D, Callon K, Porteous F, Horne A, Wattie D, et al. Imatinib promotes osteoblast differentiation by inhibiting PDGFR signaling and inhibits osteoclastogenesis by both direct and stromal cell-dependent mechanisms. J Bone Miner Res 2007;22:1679–89.
[25] Owen S, Hatfield A, Letvak L. Imatinib and altered bone and mineral metabolism. N Engl J Med 2006;355:627.
[26] Fitter S, Dewar AL, Kostakis P, To LB, Hughes TP, Roberts MM, et al. Long-term imatinib therapy promotes bone formation in CML patients. Blood 2008;111: 2538–47.
[27] Fierro F, Illmer T, Jing D, Schleyer E, Ehninger G, Boxberger S, et al. Inhibition of platelet-derived growth factor receptorβ by imatinib mesylate suppresses proliferation and alters differentiation of human mesencymal stem cells *in vitro*. Cell Prolif 2007;40:355–66.
[28] Tibullo D, Giallongo C, La Cava P, Berretta S, Stagno F, Chiarenza A, et al. Effects of imatinib mesylate in osteoblastogenesis. Exp Hematol 2009;37:461–8.
[29] Gallet M, Mentaverri R, Sevenet N, Brazier M, Kamel S. Ability of breast cancer cell lines to stimulate bone resorbing activity of mature osteoclasts correlates with an anti-apoptotic effect mediated by macrophage colony stimulating factor. Apoptosis 2006;11:1909–21.
[30] Grey A, Banovic T, Naot D, Hill B, Callon K, Reid I, et al. Lysophosphatidic acid is an osteoblast mitogen whose proliferative actions involve G(i) proteins and protein kinase C, but not P42/44 mitogen-activated protein kinases. Endocrinology 2001;142:1098–106.
[31] Groot CG, Danes JK, van der Meer JM, Herrmann-Erlee MP. Osteocalcin antigenicity in cultured osteoblast-like cells after stimulation with 1,25-vitamin D3. Cell Biol Int Rep 1985;9:528.
[32] Herrmann-Erlee MPM, van der Meer JM, editors. The effect of PTH and PGE2 on growth and differentiation of primary fetal rat osteblast-like cells, on UMR 106 osteosarcoma cells, and on a SV40 transformed "osteoblast-like" cell lineInt Conf IX on Calcium Regulating Hormones and Bone Metabolism; 1986.
[33] Cornish J, Callon KE, Lin CQ, Xiao CL, Mulvey TB, Coy DH, et al. Dissociation of the effects of amylin on osteoblast proliferation and bone resorption. Am J Physiol 1998;274:E827–33.
[34] Grey A, Chen Q, Xu X, Callon K, Cornish J. Parallel phosphatidylinositol-3 kinase and p42/44 mitogen-activated protein kinase signaling pathways subserve the mitogenic and antiapoptotic actions of insulin-like growth factor I in osteoblastic cells. Endocrinology 2003;144:4886–93.
[35] Middleton-Hardie C, Zhu Q, Cundy H, Lin JM, Callon K, Tong PC, et al. Deletion of aspartate 182 in OPG causes juvenile Paget's disease by impairing both protein secretion and binding to RANKL. J Bone Miner Res 2006;21:438–45.

[36] Walton RJ, Bijvoet OL. A simple slide-rule method for the assessment of renal tubular reaborption of phosphate in man. Clin Chim Acta 1977;81:273–6.
[37] Grey A, Bolland M, Gamble G, Wattie D, Horne A, Davidson J, et al. The peroxisome proliferator-activated receptor-gamma agonist rosiglitazone decreases bone formation and bone mineral density in healthy postmenopausal women: a randomized, controlled trial. J Clin Endocrinol Metab 2007;92:1305–10.
[38] Hofbauer LC, Khosla S, Dunstan CR, Lacey DL, Boyle WJ, Riggs BL. The roles of osteoprotegerin and osteoprotegerin ligand in the paracrine regulation of bone resorption. J Bone Miner Res 2000;15:2–12.
[39] Li B, Boast S, de los Santos K, Schieren I, Quiroz M, Teitelbaum SL, et al. Mice deficient in Abl are osteoporotic and have defects in osteoblast maturation. Nat Genet 2000;24:304–8.
